# Exploring the Risk of Alcohol Addiction and Substance Use Disorders in Darier Disease and Hailey–Hailey Disease

**DOI:** 10.2340/actadv.v105.43756

**Published:** 2025-06-25

**Authors:** William JEBRIL, Philip CURMAN, Berta OLAFSDOTTIR, Etty BACHAR-WIKSTRÖM, Jakob D. WIKSTRÖM

**Affiliations:** 1 Dermatology and Venereology Division, Department of Medicine (Solna), Karolinska Institutet, Stockholm; 2 Dermato-Venereology Clinic, Karolinska University Hospital, Stockholm; 3 Department of Medical Epidemiology and Biostatistics, Karolinska Institutet, Stockholm, Sweden

*To the Editor,*

Darier disease (DD) and Hailey–Hailey disease (HHD) are rare genodermatoses caused by mutations affecting the endoplasmic reticulum and Golgi apparatus, respectively, leading to dyshomeostasis of intracellular calcium. Both conditions are characterized by intermittent chronic lesions prone to superinfection, dysbiosis, and malodour (1), which in turn can lead to stigmatization and decreased mental well-being. Clinically, DD has long been associated with psychiatric comorbidities, with increasing evidence pointing to links with depression, anxiety, self-harm, and suicidality (2–4). HHD, on the other hand, has not demonstrated associations with psychiatric disorders, and evidence for addiction remains non-existent.

First, we conducted a nationwide register-based cohort study to investigate the co-occurrence of alcohol and substance use disorder in DD and HHD. Our study includes the Swedish National Patient Register (NPR), which utilizes the International Statistical Classification of Diseases and Related Health Problems, 10th Revision (ICD-10) code data. We identified 935 individuals with DD and 342 patients with HHD and matched them on a 1:100 ratio with non-disease controls (**Table I**). To further explore addiction and to compare DD with HHD, we conducted a questionnaire study following ethical approval from the Swedish Ethical Review Authority (case number: 2015/1798-31) involving Swedish DD and HHD cohorts at the Karolinska University Hospital in Stockholm, Sweden. We mailed validated assessment tools to 120 patients, including the Alcohol Use Disorders Identification Test (AUDIT) (5) for hazardous drinking and alcohol dependence, the Drug Use Disorders Identification Test (DUDIT) (5) for hazardous drug use and drug dependence, and the CAGE (Cut-down, Annoyance, Guilt, Eye-opener) questionnaire (6), a brief 4-item tool for alcohol-related concerns. Interpretation of questionnaire scores can be seen in Table I.

**Table I:** Epidemiological and clinical data. Epidemiological data: this incidence density sampling matching scheme allowed the odds ratios to be regarded as risk ratios (RRs)

Epidemiological data	Hailey–Hailey disease (*n* = 342) *n* (%)	Comparison group (*n* = 34,200) *n* (%)	RR (CI)	Darier disease (*n* = 935) *n* (%)	Comparison group (*n* = 93,487) *n* (%)	RR (CI)
Alcohol use disorder	8 (2.3)	958 (2.8)	0.8 (0.4;1.7)	30 (3.2)	2,348 (2.5)	1.3 (0.9;1.9)
Substance use disorder	4 (1.2)	600 (1.8)	0.6 (0.2;1.8)	32 (3.4)	1,646 (1.8)	2.0 (1.4;2.8)
Category for clinical data	Gender (*n*)	Age (years)	AUDIT (0–40 points)	CAGE (0–4 points)	DUDIT (0–44 points)	
Darier disease	17F, 11M (*n* = 28)	23–84 (mean: 55.75, median: 57)	Min: 0, max: 9, mean: 2.93	0: *n* = 20 1: *n* = 2	All patients scored 0, except 2 males who scored 4 and 5	
Hailey–Hailey disease	22F, 13M (*n* = 35)	17–85 (mean: 57.51, median: 57)	Min: 0, max: 7, mean: 2.5	0–1: *n* = 21 2: *n* = 1 3: *n* = 1	All answered 0	

RRs with corresponding 95% confidence intervals (CIs). Clinical data: AUDIT scores interpretation: 1–7 (low risk), 8–15 (hazardous drinking), and > 15 (probable dependence). DUDIT scores interpretation: 0–5 (low risk), 6–24 (moderate risk), and 25+ (high risk), with cut-offs of 6+ for males and 2+ for females indicating potential drug-related problems. The CAGE questionnaire is a brief 4-item tool where 2 or more “yes” responses indicate potential alcohol dependence and the need for further assessment.

From the NPR epidemiological data, we found that the DD patient group exhibited a doubled risk of substance use disorder (RR 2.0, 95% confidence interval [CI] 1.4; 2.8), but no increased risk of alcohol use disorder (RR 1.3, 95% CI 0.9; 1.9), while the HHD cohort did not exhibit an increased risk for alcohol use disorder or substance use disorder (Table I). In our clinical cohort, 28/60 (47%) DD patients and 35/60 (58%) HHD patients responded (Table I). The DD cohort demonstrated primarily low-risk alcohol use. AUDIT scores showed that only 1 individual scored in the “Hazardous” range (8–14) (min 0, max 9, mean 2.93). CAGE results indicated no significant alcohol-related concerns; all were below the threshold for concern (≥ 2). On the DUDIT, all participants scored below the problematic threshold, though 2 males had elevated scores, suggesting a need for closer monitoring. The HHD cohort also exhibited low-risk behaviours. AUDIT scores showed the majority of patients in the “Low-Risk” range (min: 0 max: 7, mean: 2.5). CAGE scores exhibited 1 patient with possible hazardous drinking behaviour, although in the past (score 3), and 1 with suspicious current problems (score 2), and all DUDIT scores were 0, reflecting no overwhelming current evidence of alcohol- or drug-related concerns in this group. Comparing the groups, DD exhibited slightly higher mean AUDIT scores (2.93 vs 2.5) and included 1 individual with hazardous drinking behaviour, which was absent in HHD. Additionally, 2 males in the DD cohort had elevated DUDIT scores (4 and 5), approaching the threshold for concern, while no such cases were observed in HHD.

The epidemiological and clinical data reveal a distinct pattern of increased substance use risk in DD compared with HHD patients, likely driven by the psychiatric burden associated with DD. A key strength of the epidemiological data is its ability to provide a population-level perspective in rare genodermatoses, identifying a significant doubled risk of substance use disorder in DD. In contrast, no such elevated risks were observed in the HHD cohort. Although both groups predominantly exhibit low-risk behaviours in the clinical cohort – potentially due to limited statistical power – the DD cohort’s isolated cases of elevated scores, combined with higher epidemiological risks, emphasize the need for targeted monitoring and support. These findings highlight the importance of addressing the unique psychosocial challenges faced by DD patients.

The clinical part of this study has several limitations, including reliance on self-reported questionnaires, which are susceptible to bias, and moderate response rates (47% for DD and 58% for HHD), raising concerns regarding selection bias. To conclude, DD patients show elevated substance use risk compared with Hailey–Hailey disease, tied to psychiatric comorbidities via nationwide clinical data. Despite low-risk behaviours clinically, we propose that there should be a low threshold for targeted interventions due to DD’s higher epidemiological risks.
